# Morphological chest CT changes in cystic fibrosis and massive hemoptysis

**DOI:** 10.1007/s00117-024-01266-4

**Published:** 2024-02-07

**Authors:** Martha Dohna, Hilmar Kühl, Sivagurunathan Sutharsan, Nora Bruns, Van Dai Vo Chieu, Susanne Hellms, Norman Kornemann, Michael J. Montag

**Affiliations:** 1 Department of Diagnostic and Interventional Radiology, Hannover Medical School, Carl-Neuberg-Str. 1, 30625 Hannover, Germany; 2 Clinic for diagnostic and interventional Radiology, University Hospital Bonn, Bonn, Germany Venusberg-Campus 1, 53127; 3 St. Bernhard-Hospital Kamp-Lintfort, Bürgermeister-Schmelzing-Str. 90, 47475 Kamp-Lintfort, Germany; 4 Division of Cystic Fibrosis, Department of Pulmonary Medicine, University Medicine Essen -Ruhrlandklinik, University of Duisburg-Essen, Essen, Germany; 5 Department of Pediatrics I, University Medicine Essen, Hufelandstr. 55, 45147 Essen, Germany; 6 St. Vicenz-Hospital Paderborn, Am Busdorf 2, 33098 Paderborn, Germany; 7 Clinic for Radiology, University Hospital Münster, Albert-Schweitzer-Campus 1, 48149 Münster, Germany

**Keywords:** Morphological lung changes, Helbich scoring system, Risk factor, Chest CT, Bronchial artery, Morphologische Lungenveränderungen, Helbich-Scoringsystem, Risikofaktor, Thorax-Computertomographie, Bronchialarterie

## Abstract

**Background:**

Massive hemoptysis (MH) is a rare but potentially life-threatening condition of patients with mainly advanced cystic fibrosis (CF). Morphological lung changes are aggravated with disease progression. The aim of this study was to determine whether morphological lung changes differ between patients with CF (pwCF) who have MH and pwCF without MH.

**Methods:**

Chest computed tomography (CT) scans of pwCF and MH acquired at a maximum of 4 months prior to MH (1/2008 to 2/2015) were evaluated for morphological changes and bronchial artery (BA) diameters. Lung lobes with MH were compared with lobes without MH and with matched control patients with end-stage CF and no hemoptysis using the Helbich scoring system.

**Results:**

The study included 26 patients with MH (P_MH_; 15 female, median age 29 years, interquartile range [IQR]: 25–33.75) and 17 matched control patients (11 male, median age 24 years, IQR: 19.5–30). No difference in Helbich score was detected between lobes with MH and matched control patients (*p* = 0.051). Higher scores were detected in lobes with MH compared to lobes without MH in P_MH_ (*p* = 0.021), but no difference was detected in the subscores. The BA diameters were larger in P_MH_ (*p* = 0.02); 85% of P_MH_ had unilateral MH, with 65% of MH involving only one or two lobes.

**Conclusion:**

Morphological changes are more severe in lobes with MH in the same patient, but there is no difference when compared with matched control patients. Besides abscess/sacculation, no specific changes for MH were identified. Other factors such as BA hypertrophy might play a pivotal role in the pathogenesis of MH in pwCF. Commonly used scores to evaluate chest CT in pwCF cannot be used to assess MH, and other factors, e.g., hypertrophied BA, not represented and not measured in these scores, might be more suitable for assessing the risk for MH.

## Abstract (de)

**Hintergrund:**

Massive Hämoptysen (MH) sind selten, aber bei Patienten mit vorwiegend fortgeschrittener zystischer Fibrose (CF) potenziell lebensbedrohend. Die morphologischen Lungenveränderungen verschlimmern sich mit dem Krankheitsfortschritt. Ziel der vorliegenden Studie war es zu untersuchen, ob die morphologischen Lungenveränderungen sich zwischen Patienten mit CF (pwCF), bei denen MH bestehen, und pwCF ohne MH unterscheiden.

**Methoden:**

Thorax-Computertomographie(CT)-Aufnahmen von pwCF mit MH, die maximal 4 Monate vor den MH (1/2008 bis 2/2015) angefertigt worden waren, wurden in Bezug auf morphologische Veränderung und Durchmesser der Bronchialarterien (BA) ausgewertet. Die Lungenlappen mit MH wurden verglichen mit Lungenlappen ohne MH und mit gematchten Kontrollpatienten mit CF im Endstadium, aber ohne Hämoptysen gemäß dem Helbich-Scoringsystem.

**Ergebnisse:**

In die Studie wurden 26 Patienten mit MH (P_MH_; 15 weiblich, Durchschnittsalter 29 Jahre, Interquartilsabstand [IQR]: 25–33,75) und 17 gematchte Kontrollpatienten eingeschlossen (11 männlich, Durchschnittsalter 24 Jahre, IQR: 19,5–30). Es wurde kein Unterschied im Helbich-Score zwischen Lungenlappen mit MH und gematchten Kontrollpatienten festgestellt (*p* = 0,051). Höhere Scores fanden sich bei Lungenlappen mit MH im Vergleich zu Lungenlappen ohne MH bei P_MH_ (*p* = 0,021), aber in den Subscores war kein Unterschied festzustellen. Die BA-Durchmesser waren bei P_MH_ größer (*p* = 0,02); 85% der P_MH_ wiesen unilateral eine MH auf, dabei betrafen 65% der MH nur einen oder 2 Lappen.

**Schlussfolgerung:**

Innerhalb desselben Patienten mit MH sind die morphologischen Veränderungen in den Lungenlappen mit MH schwerer ausgeprägt, aber es besteht kein Unterschied, wenn man sie mit gematchten Kontrollpatienten vergleicht. Außer Abszess/Sakkulation wurden keine spezifischen Veränderungen für MH festgestellt. Andere Faktoren, wie eine BA-Hypertrophie, könnten eine entscheidende Rolle in der Pathogenese von MH bei pwCF spielen. Häufig verwendete Scores zur Auswertung einer Thorax-CT bei pwCF können nicht zur Beurteilung von MH eingesetzt werden, und andere Faktoren, z. B. hypertrophierte BA, die nicht in diesen Scores repräsentiert und gemessen werden, könnten zur Einschätzung des Risikos von MH besser geeignet sein.

## Introduction

Patients with cystic fibrosis (pwCF) experience massive hemoptysis (MH) as a rare but potentially life-threatening complication with mortality rates as high as 75% [1, 2]. Overall, 1–4.1% of pwCF will experience MH with an annual incidence of approximately 1% [3]. Commonly, older patients with advanced disease are affected, but also patients with only minor impairment of lung function and even children can experience MH [3–5]. Many pwCF report blood-stained sputum and/or recurrent minor hemoptysis, which is in most cases self-limiting and does not require therapy [6, 7]. However, when MH occurs, it may create a medical emergency demanding immediate therapy to save the patient [3, 8]. Medical emergencies are often carried out in suboptimal conditions, perhaps at night and with reduced staff, patients must be transferred to a specialized center for bronchial artery embolization (BAE), and, equally important, the patient is likely to be in a severely compromised and unstable condition, which negatively influences outcome.

Cystic fibrosis is a chronic disease causing local and systemic inflammation that results in a variety of structural lung tissue changes over time. Upregulation of serum vascular endothelial growth factor is triggered by this inflammatory process promoting hypertrophy of bronchial arteries (BAs), which become enlarged, tortuous, and dilated vessels [9]. The BAs are localized submucosally in the bronchial wall. Flume et al. hypothesize that chronic and acute inflammation weakens the vessel walls and can lead to episodic or persistent bleeding into the bronchial lumen, with subsequent hemoptysis [3, 10]. According to this hypothesis, it would be expected that massive hemoptysis would occur in patients with more severe disease, yet the complication has been known to occur in patients with seemingly milder disease [3, 5, 11, 12].

Surveillance of lung tissue changes is possible with chest x‑ray, computed tomography scans (CT), commonly non-contrast-enhanced, and increasingly with magnetic resonance imaging (MRI), as MRI protocols and imaging quality are improving [13, 14]. Medical imaging is evaluated for morphological lung tissue changes and an overall point score of the lung is created to compare examinations over time and objectify disease progression. For the evaluation of chest CT scans in pwCF, different morphology scoring systems are applied, e.g., the Helbich score [15–18]. However, the existing scoring systems do not consider the anatomical distribution of morphological changes, nor do they take into account the side or separate lobes affected by MH. Although bronchiectasis has been described as a main risk factor for MH [19–21], data on risk factors for MH in CF are scarce [22–24] and data evaluating morphological changes of chest CT in pwCF and MH do not exist.

The aim of this study was to identify differences in morphological changes in lobes affected by MH and lobes not affected by MH in the same patient in comparison with patients who have end-stage CF but without MH, using the Helbich scoring system for chest CT assessment [16].

## Material and methods

### Patients with MH

Patients or public were not involved in the design, conduct, reporting, or dissemination plans of our research. This study is a secondary subsample analysis (*n* = 26) of a previously published multicenter study carried out between January 2008 and February 2021 measuring BA diameters in pwCF [24]. In the present study, morphological changes in pwCF and MH were evaluated, which was not part of the parent study.

All adult pwCF referred to our hospital for MH between January 2008 and February 2015 who underwent at least one super-selective BA coil embolization (ssBACE) were included in this retrospective multicenter study. Patients arrived at our institution with external chest CT scans from ten different hospitals across Germany. The CT scans therefore differed in terms of protocols and scanning devices. The gender and age of the patients, forced expiratory volume in 1 s in % predicted (ppFEV_1_), coagulation parameters, pulmonary exacerbation at the time of MH, and intervention data were documented. For all patients, the attending hospitals were contacted again in 2022 for follow-up.

If patients underwent more than one ssBACE procedure, the CT scans refer to the first intervention. Localization of the culprit lobe(s) was based on the patient’s perception and on digital subtraction angiography findings, and considered correct if hemostasis was obtained for at least 48 h [10, 25, 26]. Here, MH was defined as > 240 ml in 24 h or recurrent bleeding of substantial volume (> 100 ml/day) for several days or weeks [22, 26]. We followed a restrictive intervention protocol embolizing suspected culprit vessels only and treating a maximum of three lobes per intervention.

### Control group

In total, 35 chest CT scans (dual-source CT scanner SOMATOM Force, Siemens, Erlangen, Germany) of patients with end-stage CF and no history of hemoptysis (P_control_1_) were evaluated retrospectively for morphological changes and BA diameter. We generated a subgroup of less affected and thus severity-matched control group patients (P_control_2_) for better comparison with P_MH_. The CT scans had been carried out between September 2007 and July 2021 to evaluate suitability for lung transplantation in all control patients. The gender, age of the patients, and ppFEV_1_ were documented.

### CT evaluation for morphological changes

All 26 CT scans of P_MH_ were contrast enhanced. All CT scans in control groups 1 and 2 were carried out as CT angiography. Chest CT scans of P_MH_ and P_control_1_ and P_control_2_ were assessed semi-quantitatively by consensus by two radiologists with 11 and 25 years of experience in chest CT (MD, HK) according to the Helbich scoring system [16]. Readers were not involved in BAE. Good intra- and interobserver agreement using this scoring system has been published previously [15, 27, 28]. Readers were blinded to patient data, the presence of hemorrhage, and the site of hemorrhage/ssBACE.

The Helbich scoring system was modified to segments per lobe instead of lobes per lung for parameters scored by the number of subunits involved (Table 1). The CT scans were evaluated for a total of 11 subscores (Table 2). A maximum of 30 points could be reached per lobe. Scores were calculated (a) as median for the right and left lung and (b) as median for lobes affected by MH and lobes not affected by MH. The lingula was counted as a separate lobe. Air trapping was not evaluated, as images in expiration had not been acquired. The BA diameters were measured in all contrast-enhanced CT scans in the mediastinum close to their origin in perpendicular orientation to the vessel.

**Table 1:** Point score of lobes with massive hemoptysis based on anatomical lung segments using the modified Helbich CT scoring system

1 Point	2 Points	3 Points
1 of 3 segments (33%)	1 of 2 segments (50%)	2 of 2 segments (100%)
1 of 4 segments (25%)	2 of 3 segments (67%)	3 of 3 segments (100%)
1 of 5 segments (20%)	2 of 4 segments (50%)	3 of 4 segments (75%)
	2 of 5 segments (40%)	4 of 4 segments (100%)
	3 of 5 segments (60%)	4 of 5 segments (80%)
		5 of 5 segments (100%)

**Table 2:** Median lobar Helbich underscores of patients with massive hemoptysis (P_MH_)

Helbich underscore of patients with MH (*n* = 26) of lobes with hemorrhage	Right UL, median (IQR, 1–3)	ML, median (IQR, 1–3)	Right LL, median (IQR, 1–3)	Left UL, median (IQR, 1–3)	Lingula, median (IQR, 1–3)	Left LL, median (IQR, 1–3)
Severity of bronchiectasis	2 (1–2)	1 (1–2)	1 (1–2)	1 (1–2)	1 (1–2)	1 (1–1.75)
Bronchial wall thickening	1 (1–2)	1 (1–1)	1 (1–1)	1 (1–2)	1 (1–1)	1 (1–1)
Extent of bronchiectasis, no. of segments	3 (3–3)	3 (3–3)	3 (3–3)	3 (3–3)	3 (3–3)	3 (3–3)
Extent of mucus plugging, no. of segments	3 (3–3)	3 (2–3)	3 (1.25–3)	3 (2–3)	3 (2–3)	3 (2–3)
Sacculation/abscess, no. of segments	0 (0–0.75)	0 (0–0)	0 (0–0)	0 (0–0.75)	0 (0–0)	0 (0–0)
Generations of bronchial divisions involved in bronchiectasis/mucus plugging	3 (2–3)	3 (2–3)	3 (3–3)	3 (3–3)	3 (2.25–3)	3 (2.25–3)
Number of bullae	0 (0–1)	0 (0–0)	0 (0–0)	0 (0–0)	0 (0–0)	0 (0–0)
Emphysema, no. of segments	0 (0–1.5)	0 (0–0)	0 (0–2)	0 (0–1.75)	0 (0–0)	0 (0–2.75)
Collapse consolidation	0 (0–1)	0 (0–0)	0 (0–0)	0 (0–0)	0 (0–0)	0 (0–0)
Mosaic attenuation, perfusion pattern	0 (0–0.0)	0 (0–0)	0 (0–1)	0 (0–0)	0 (0–1.5)	0 (0–2)
Ground glass infiltrate	0 (0–1)	0 (0–0)	0 (01)	0 (0–0)	0 (0–0)	0 (0–0)

*MH* massive hemoptysis, *no.* number, *UL* upper lobe, *ML* middle lobe, *LL* lower lobe, *IQR* interquartile range

### Statistical analysis

Data were highly skewed and are therefore presented as median/interquartile range. The Mann–Whitney *U *test for two independent groups was used to compare the Helbich scores of lung lobes with MH against lobes without MH in the same patient and against P_control_1_ and P_control_2_, and to calculate differences in BA diameters in P_MH_, P_control_1_ and P_control_2_. The Kruskal–Wallis test was used to compare the Helbich subscores in P_MH_, P_control_1_ and P_control_2_. Bonferroni correction of data was performed to adapt the significance levels. Statistical analysis was carried out using IBM SPSS Statistics Version 27. Values of *p* < 0.05 were considered statistically significant.

### Ethics approval and consent to participate

This retrospective multicenter study has been approved by the ethics committee of the Aerztekammer Nordrhein according to § 15 registry number 2016/181 and by the ethics committee of the Medical School Hannover, registry number 10245_BO_K_2022, who also waived the need for informed consent from all patients. All methods were carried out in accordance with relevant guidelines and regulations.

## Results

### Patients with massive hemoptysis

A total of 34 consecutive pwCF who had undergone ssBACE for MH in our institution between January 2008 and February 2015 met the inclusion criteria of this study. Four patients had to be excluded as no CT scan prior to ssBACE had been carried out due to the urgency of the situation. Four patients had to be excluded because the time of the CT scan exceeded the 4‑month interval prior to intervention. Overall, 26 patients (15 female, median age 29 years, IQR: 25–33.75) had received CT scans 0 days to 4 months (IQR 9 days, 1.25–25.5) prior to embolization and were included for CT morphology evaluation and BA measurements. In seven patients, CT scans were carried out 0–1 day prior to MH. The intervention characteristics of P_MH_ are listed in Table 3. The median ppFEV_1_ was 46.5% (37–63.7%). Coagulation parameters were normal in all patients. Eight patients (31%) died during the observation period 2008–2022. In the patient charts exacerbation of pulmonary infection was noted as “severe pulmonary infection” at time of hemoptysis in three cases and as “no current infection” in four cases. In the remaining 19 patients no information concerning pulmonary exacerbation at the time of MH was available, which led to the exclusion of pulmonary exacerbation for statistical analysis. In 17 patients (65%) MH occurred during the cold season in Germany from October to April. In two of three cases with pulmonary exacerbation, MH occurred during the cold season. In three of five cases without pulmonary exacerbation, MH occurred outside the cold season.

**Table 3:** Intervention characteristics of bronchial artery embolization in patients with massive hemoptysis

Intervention characteristics	Patients, *n* = 26	Patients in %
Right UL affected by MH	14	54
ML affected by MH	8	31
Right LL affected by MH	9	35
Left UL affected by MH	8	31
Lingula affected by MH	4	15
Left LL affected by MH	5	19
*Unilateral BAE*	22	85
Right lung	15	58
Left lung	7	27
*BAE in one single lobe*	11	42
Only right lung	7	27
Only left lung	4	15
*BAE in two lobes*	6	23
Only right lung	4	15
Only left lung	2	8
*BAE in three lobes*	7	27
Only right lung	4	15
Only left lung	1	4
Reintervention during 7‑year observation period	14	54
Survival during 7‑year observation period	18	69

*UL* upper lobe, *ML* middle lobe, *LL* lower lobe, *MH* massive hemoptysis, *BAE* bronchial artery embolization

### Control groups

In total, 35 P_control_1_ (21 male, median age 24.0 years, IQR, 18.5–33.5, ppFEV_1_ 30% [25–43.5]) were included as control group patients P_control_1_. A total of 17 patients (11 male, median age 24 years (19.5–30), ppFEV_1_ 45% [32–55]) were included in the severity-matched subgroup P_control_2._

### Morphological CT scan evaluation

#### Helbich score

The median modified Helbich score in P_MH_ including all lobes was lower with 76.5 points (67.25–88.5), than in control group 1 patients (P_control_1_) with 98 points (79–110), and comparable to the severity-matched control group 2 (P_control_2_) with 76 points (69–92). There was no difference in median Helbich score of the right lung at 39 points (34.25–45.25) compared to the left lung with 39 points (33–46.5) in P_MH_ (*p* = 1.0), and neither in P_control_1_ with 50 points (IQR, 41–56) for the right lung and 48 points (IQR, 38.5–53) for left lung (*p* = 1.0) nor in P_control_2_ with 40 points (35–45) for the right lung compared to 37 points (35–43) for the left lung (*p* = 1.0). In patients with unilateral MH (*n* = 22), the median Helbich point score for lungs with MH was 39 points (IQR, 33.25–42.75) and 39 points (IQR 33–44.75) of lungs not affected by MH (*p* = 0.75). The median lobar score of P_MH_ was 13 (11–16). In P_MH_, the median lobar score of all lobes with MH was 14 (12–16), and the median lobar score of all lobes without MH was 13 (11–15; *p* = 0.021). The median lobar score of P_control_1_ was 16 (13–18) and thus higher than in lobes with MH (*p* = 0.004). The median lobar score in P_control_2_ was 13 (11–15) and there was no difference compared to lobes with MH (*p* = 0.051) and compared to P_MH_ (*p* = 0.87). The median subscores “severity of bronchiectasis” (*p* = 0.34) and “bronchial wall thickening” (*p* = 0.122) were both higher in lobes with MH than in lobes without MH but this did not reach statistical significance. The severity of bronchiectasis was significantly lower in lobes with MH compared to P_control_1_ (*p* = 0.001; Table 4) and P_control_2_ (*p* = 0.003; Table 5). The subscore “ground glass infiltrate” was significantly lower in P_MH_ than in P_control_1_ (*p* = 0.01) but not in P_control_2_ (*p* = 1.0) and there was no difference in lobes with MH compared to P_control_1_ (*p* = 0.14) and to P_control_2_ (*p* = 1.0). In the seven CT scans carried out directly at the time of MH, this subscore showed a distribution independent of lobes with MH in all but one patient. The subscore “emphysema” was higher in P_control_1_ than in lobes with MH (*p* = 0.09) and P_MH_ (*p* = 0.07), but no difference was detected in lobes with MH compared to lobes without MH (*p* = 1.0) and to P_control_2_ (*p* = 1.0). The subscore “sacculation/abscess” was significantly higher in lobes with MH than in P_control_2_ (*p* = 0.05) but there was no difference when compared to P_control_1_ (*p* = 0.18), and no difference was found between lobes with MH and lobes without MH in P_MH_ (*p* = 0.23; Table 4). The subscore “generations of bronchial divisions involved in bronchiectasis/mucus plugging” was higher in P_control_1_ than in lobes with MH (*p* = 0.07) but there was no difference between lobes with and without MH (*p* = 1.0) and there was also no difference between lobes with MH and P_control_2_ (*p* = 0.51). All other Helbich subscores did not show significant differences, neither between lobes with MH and without MH in P_MH_ nor compared to P_control_1_ or P_control_2_. The median Helbich subscores of lobes with and without MH in P_MH_, P_control_1_, and P_control_2_ are listed in Tables 4 and 5. The median Helbich lobar subscores are shown in Table 2 for P_MH_, in Table 6 for P_control_1_, and in Table 7 for P_control_2_.

**Table 4:** Median Helbich subscores in patients with MH and in control group patients (P_control_1_)

Helbich subscores	Median point score of lobes with MH (IQR, 1–3)	Median point score of lobes without MH (IQR, 1–3)	Difference between lobes with MH and lobes without MH	Median point score of all lobes in P_MH_ (IQR, 1–3)	Difference between Lobes with MH and P_control_1_	Median point score of P_control_1_ (IQR, 1–3)	Difference between P_MH_ and P_control_1_
Severity of bronchiectasis	1.5 (1–2)	1 (1–1)	*p* = 0.34	1.5 (1–1.5)	*p* = 0.001	3(3–3)	*p* = 0.001
Bronchial wall thickening	1 (1–2)	1 (1–1)	*p* = 0.12	1 (1–1)	*p* = 1.0	1(1–1.5)	*p* = 1.0
Extent of bronchiectasis, no. of segments	3 (3–3)	3 (3–3)	*p* = 1.0	3 (3–3)	*p* = 1.0	3(3–3)	*p* = 1.0
Extent of mucus plugging, no. of segments	3 (1.6–3)	3 (2–3)	*p* = 1.0	3 (2.13–3)	*p* = 1.0	3(2.5–3)	*p* = 1.0
Sacculation/abscess, no. of segments	0 (0–0.4)	0 (0–0)	*p* = 0.23	0 (0–0)	*p* = 0.18	0(0–0)	*p* = 1.0
Generations of bronchial divisions involved in bronchiectasis/mucus plugging	3 (2.6–3)	3 (3–3)	*p* = 1.0	3 (3–3)	*P* = 0.18	3(3–3)	*p* = 0.07
Number of bullae	0 (0–0)	0 (0–0)	*p* = 0.31	0 (0–0)	*p* = 1.0	0(0–0)	*p* = 1.0
Emphysema, no. of segments	0 (0–1.75)	0 (0–1.13)	*p* = 1.0	0 (0–1.63)	*p* = 0.09	2(0–3)	*p* = 0.07
Collapse, consolidation	0(0–1)	0 (0–0)	*p* = 1.0	0 (0–0)	*p* = 0.65	0(0–0)	*p* = 1.0
Mosaic attenuation, perfusion pattern	0 (0–0)	0 (0–0.75)	*p* = 1.0	0 (0–0)	*p* = 1.0	0(0–1)	*p* = 1.0
Ground glass infiltrate	0 (0–0.75)	0 (0–0)	*p* = 1.0	0 (0–0.75)	*p* = 0.14	1(0–2)	*p* = 0.01

*IQR* interquartile range, *MH* massive hemoptysis, *no.* number, *IQR* interquartile range, *P*_*MH*_  patients with massive hemoptysis, *P*_*control_1*_  control patients without hemoptysis

**Table 5:** Median Helbich subscores in patients with MH and in matched control group patients P_control_2_

Helbich subscores	Median point score of lobes with MH (IQR, 1–3)	Median point score of lobes without MH (IQR, 1–3)	Median point score of all lobes in P_MH_ (IQR, 1–3)	Median point score P_control_2_ (IQR, 1–3)	Difference between lobes with MH and P_control_2_	Difference between P_MH_ and P_control_2_
Severity of bronchiectasis	1.5(1–2)	1 (1–1)	1.5 (1–1.5)	3 (3–3)	*p* = 0.003	*p* = 0.001
Bronchial wall thickening	1 (1–2)	1 (1–1)	1 (1–1)	1 (1–1)	*p* = 0.45	*p* = 1.0
Extent of bronchiectasis, no. of segments	3 (3–3)	3 (3–3)	3 (3–3)	3 (3–3)	*p* = 1.0	*p* = 1.0
Extent of mucus plugging, no. of segments	3 (1.6–3)	3 (2–3)	3 (2.13–3)	3 (3–3)	*p* = 1.0	*p* = 1.0
Sacculation/abscess, no. of segments	0 (0–0.4)	0 (0–0)	0 (0–0)	0 (0–0)	*p* = 0.05	*p* = 1.0
Generations of bronchial divisions involved in bronchiectasis/mucus plugging	3 (2.6–3)	3 (3–3)	3 (3–3)	3 (3–3)	*p* = 0.94	*p* = 0.51
Number of bullae	0 (0–0)	0 (0–0)	0 (0–0)	0 (0–0)	*p* = 1.0	*p* = 1.0
Emphysema, no. of segments	0 (0–1.75)	0 (0–1.13)	0 (0–1.63)	0 (0–2)	*p* = 1.0	*p* = 1.0
Collapse, consolidation	0 (0–1)	0 (0–0)	0 (0–0)	0 (0–0)	*p* = 0.24	*p* = 1.0
Mosaic attenuation, perfusion pattern	0 (0–0)	0 (0–0.75)	0 (0–0)	0 (0–0.5)	*p* = 1.0	*p* = 1.0
Ground glass infiltrate	0 (0–0.75)	0 (0–0)	0 (0–0.75)	0 (0–1.38)	*p* = 1.0	*p* = 1.0

*IQR* interquartile range, *MH* massive hemoptysis, *no*. number, *IQR* interquartile range, *P*_*MH*_  patients with massive hemoptysis, *P*_*control_2*_  matched control patients without hemoptysis

**Table 6:** Median lobar Helbich subscores of control patients without hemoptysis (P_control_1_)

Helbich underscore of control group 2 (no hemoptysis) (*n* = 35)	Right UL median (IQR, 1–3)	ML median (IQR, 1–3)	Right LL median (IQR, 1–3)	Left UL median (IQR, 1–3)	Lingula median (IQR, 1–3)	Left LL median (IQR, 1–3)
Severity of bronchiectasis	2 (1–3)	2 (1–2)	1 (1–2)	2 (1–3)	2 (1–2)	1 (1–2)
Bronchial wall thickening	1 (1–2)	1 (1–2)	1 (1–1)	1 (1–1)	1 (1–2)	1 (1–1)
Extent of bronchiectasis, no. of segments	3 (3–3)	3 (3–3)	3 (3–3)	3 (3–3)	3 (3–3)	3 (3–3)
Extent of mucus plugging, no. of segments	3 (3–3)	3 (2–3)	3 (3–3)	3 (2.5–3)	3 (3–3)	3 (2–3)
Sacculation/abscess, no. of segments	0 (0–0)	0 (0–0)	0 (0–0)	0 (0–0)	0 (0–0)	0 (0–0)
Generations of bronchial divisions involved in bronchiectasis/mucus plugging	3 (3–3)	3 (3–3)	3 (3–3)	3 (3–3)	3 (3–3)	3 (3–3)
Number of bullae	0 (0–1)	0 (0–0)	0 (0–0)	0 (0–0)	0 (0–0)	0 (0–0)
Emphysema, no. of segments	2 (0–3)	2 (0–3)	2 (0–3)	2 (0–3)	2 (0–3)	2 (0–3)
Collapse consolidation	0 (0–1)	0 (0–1.5)	0 (0–0)	0 (0–0)	0 (0–0)	0 (0–0)
Mosaic attenuation, perfusion pattern	0 (0–1.5)	0 (0–0)	0 (0–2)	0 (0–1.5)	0 (0–0)	0 (0–2)
Ground glass infiltrate	1 (0–3)	0 (0–2)	1 (0–2.5)	1 (0–3)	1 (0–2)	1 (0–2.5)

*MH* massive hemoptysis, *no.* number, *UL* upper lobe, *ML* middle lobe, *LL* lower lobe, *IQR* interquartile range

**Table 7:** Median lobar Helbich subscores of matched control patients without hemoptysis (P_control_2_)

Helbich underscore of control group 2 (no hemoptysis) (*n* = 17)	Right UL median (IQR, 1–3)	ML median (IQR, 1–3)	Right LL median (IQR, 1–3)	Left UL median (IQR, 1–3)	Lingula median (IQR, 1–3)	Left LL median (IQR, 1–3)
Severity of bronchiectasis	1.5 (1–2)	1 (1–2)	1 (1–1)	1 (1–2)	1 (1–2)	1 (1–1)
Bronchial wall thickening	1 (1–1.75)	1 (1–1.75)	1 (1–1)	1 (1–1)	1 (1–1)	1 (1–1)
Extent of bronchiectasis, no. of segments	3 (3–3)	3 (3–3)	3 (3–3)	3 (3–3)	3 (3–3)	3 (3–3)
Extent of mucus plugging, no. of segments	3 (1–3)	2 (0.5–3)	3 (1–3)	3 (1.25–3)	2 (1–3)	3 (2–3)
Sacculation/abscess, no. of segments	0 (0–0)	0 (0–0)	0 (0–0)	0 (0–0)	0 (0–0)	0 (0–0)
Generations of bronchial divisions involved in bronchiectasis/mucus plugging	3 (3–3)	3 (3–3)	3 (3–3)	3 (3–3)	3 (3–3)	3 (3–3)
Number of bullae	0 (0–1.75)	0 (0–0)	0 (0–0)	0 (0–0)	0 (0–0)	0 (0–0)
Emphysema, no. of segments	0 (0–2)	0 (0–2)	0 (0–2)	0.5 (0–2)	0 (0–2)	0 (0–2)
Collapse consolidation	0 (0–0)	0 (0–0)	0 (0–0)	0 (0–0)	0 (0–0)	0 (0–0)
Mosaic attenuation, perfusion pattern	0 (0–1)	0 (0–0)	0 (0–1)	0 (0–1.5)	0 (0–0)	0 (0–1.75)
Ground glass infiltrate	1 (0–3)	0 (0–0.75)	0 (0–1.75)	0.5 (0–2)	0.5 (0–2)	1 (0–1.75)

*MH* massive hemoptysis, *no*. number, *UL* upper lobe, *ML* middle lobe, *LL* lower lobe, *IQR* interquartile range

In P_MH_, “severity of bronchiectasis” was most marked in the right upper lobe and the left lower lobe in lobes with MH (Table 2). The maximum lobar score was reached for the right upper and left lower lobe in lobes with MH and in the left upper lobe in lobes without MH (Table 8). In P_control_1_ and P_control_2_, both upper lobes reached the highest median lobar point scores (Table 8).

**Table 8:** Median lobar scores in patients with MH and control group 1 and 2 patients

Patients	Right UL median (IQR, 1–3)	ML median (IQR, 1–3)	Right LL median (IQR, 1–3)	Left UL median (IQR, 1–3)	Lingula median (IQR, 1–3)	Left LL median (IQR, 1–3)
Patients with MH, lobes with MH *N* = 26	16 (15–17)	13 (11.75–14.25)	13 (12–15)	15 (11.75–16.25)	12 (11.75–13.25)	18.5 (13–16)
Patients with MH, lobes without MH *N* = 26	13 (11.75–17)	11 (8–15)	12 (11–14)	14 (11.25–16)	13.5 (10.25–14.75)	13 (8–14)
Control group 1 without hemoptysis *N* = 35	17 (15–19)	16 (13–18)	16 (12–18.5)	17 (14–18)	15 (13–17)	15 (12–17)
Matched control group 2 without hemoptysis *N* = 17	15 (12–17)	13 (11–15)	11 (11–16)	14 (12–15)	13 (12–14)	12 (11–15)

*MH* massive hemoptysis, *UL* upper lobe, *ML* middle lobe, *LL* lower lobe, *IQR* interquartile range

#### Bronchial artery diameters

The BA diameter measurements in P_MH_ showed a median of 3.9 mm (3.3–4.3) and were higher than BA diameters of P_control_1_ with a median of 3.2 mm (2.6–3.7) (*p* = 0.008) and higher than BA diameters in P_control_2_ with 3.0 mm (2.6–3.6) (*p* = 0.02).

## Discussion

This retrospective multicenter study on chest CT findings in pwCF found no difference in the severity of morphological changes in lobes with MH in patients with MH when compared to control patients without MH. However, median lobar scores were higher in lobes with MH compared to lobes without MH in the same patient but without any difference when compared with severity-matched control patients. The subscore “sacculation/abscess” was higher in lobes with MH compared to severity-matched control patients but with overlapping interquartile ranges and without any difference when compared with lobes without MH. The subscores “severity of bronchiectasis,” “emphysema,” “ground glass opacity,” and “generations of bronchial divisions involved in bronchiectasis/mucus plugging” were significantly higher in P_control_1_ and P_control_2_ than in P_MH_ and in lobes with MH. None of the subscores showed significant differences between lobes with MH and lobes without MH. The BA diameters were significantly higher in P_MH_ than in both control groups.

Although there is common agreement on BAs being the culprit vessels in MH, there is debate about why and when and in whom BAs rupture or erode and cause MH [3, 12, 25]. The results of our study put into question the hypothesis of severe morphological changes inducing MH in pwCF. Prevailing theories for the development of hemoptysis suggest combined inflammatory and mechanical stress weakens the walls of hypertrophied and friable submucosal BAs, which then erode and bleed into the airways [3, 10]. This hypothesis seemed corroborated by the fact that MH occurred mainly in advanced disease with major morphological lung changes [1, 3, 8, 29]. Thompson et al. described older age and more severe pulmonary impairment as risk factors for MH [29].

In our cohort we found morphological changes predominantly in the upper lobes and in the right lung, a common finding in CF [18], with equal distribution in P_MH_ and in both control groups. Furthermore, MH also predominantly occurred in the right lung, in the upper lobes, and with older age in our cohort, as was also described in the literature [3, 4, 23]. On the other hand, hemoptysis and MH also occur in young patients and children as well as in patients with well-preserved lung function independent of the severity of lung disease [5, 12].

In our study, no difference was detected in total lung score of P_MH_ compared to P_control_1_ nor compared to the severity-matched control group P_control_2_using a common scoring system. Moreover, when evaluated separately for lungs with and lungs without hemoptysis in patients with unilateral MH, no difference in point score was found. When evaluated on a lobar level, the median lobar score in lobes with MH was not higher than in the severity-matched P_control_2_. However, median lobar scores in lobes with MH were significantly higher than in lobes without MH, indicating that morphological changes contribute at least in part to MH, but no specific subscores were identified. The subscore “severity of bronchiectasis” was significantly less severe in P_MH_ and in lobes with MH than in both control groups that did not suffer from MH. This is especially interesting as bronchiectasis has been described as a main risk factor for MH in a variety of lung conditions [19–21].

Contrary to common practice, which includes treating all enlarged BAs, we followed a protocol of prudent embolization, exclusively treating lobes affected by MH. This enabled us to identify lobes with MH and lobes without MH for scoring. However, this information is not available from routine chest CT. But as most patients are able to localize the bleeding site [10, 25, 26], this important clinical information might be integrated in assessing the risk for MH together with chest CT findings. For the clinician to use chest CT evaluation for MH risk estimate, it would be necessary to carry out chest CT interpretation on a lobar level and to identify lobes with the highest score or rapid progression in the same patient. However, the hypothesis of morphological changes correlating with or even causing MH might be reconsidered, since morphological changes were not more severe in lobes with MH compared to P_control_2_ with comparable global Helbich scores as P_MH_.

Exacerbation of pulmonary infection has been described to be associated with MH [10, 23, 30]. Efrati et al. described exacerbation of a pulmonary infection as the most common precipitating factor for a hemoptysis event in 90% of study patients [5]. It was found that MH occurred more often during winter months when infections are more common [23]. In our cohort, data concerning pulmonary exacerbation from patient charts were too scarce for statistical evaluation, but MH occurred during the cold season in 65% of patients. Interestingly, the subscore “ground glass infiltrate” was significantly more marked in P_control_1_ than in P_MH_, but no difference was noted between lobes with and lobes without MH, also when compared to P_control_2_. Repetitive aspiration of coughed-up blood might lead to the redistribution of intraluminal blood to various lobes possibly explaining why the subscore “ground glass infiltrate,” often seen as a correlate of hemoptysis, did not show higher scores in lobes with MH than in lobes without MH. This higher subscore in P_control_1_ but not in P_control_2_ might be explained by more advanced disease in P_control_1_. Also in line with this finding, the subscore “consolidation,” a correlate for pulmonary infection, did not show any difference between P_MH_ and control patients nor between lobes affected by MH and lobes not affected by MH. We believe more severe findings in control patients reflect more severe disease. Of note, the subscore “abscess/sacculation” was higher in lobes with MH compared to P_control_2_, but not higher than in lobes without MH. However, this subscore should be considered as a possible risk factor for MH, especially when found in an area targeted by the patient for hemoptysis. Interestingly, 65% of patients undergoing ssBACE required intervention in only one or two lobes. In 85% of patients, MH was unilateral and 50% of these patients underwent a single-lobe intervention.

These findings indicate that MH is, at least in the majority of patients, a localized process not reflected in common scoring systems. Although morphological CT changes and MH occur in the same region and in pwCF with older age, CT morphology assessed with the Helbich score, or other common scores [15–18], does not seem to reflect bleeding risk, as the global morphology score and several subscores were highest in P_control_1_. Severe morphological changes and changes leading to MH might both progress over time, possibly both triggered by chronic inflammation, but they might not be linked and might not induce each other, instead constituting parallel processes. Data on possible factors causing MH are scarce and the pathogenesis of MH is yet unknown [3]. Presumably, the presence of other factors such as significant BA hypertrophy, which is not automatically found in patients with advanced disease, or the presence of *Staphylococcus aureus,* might play a role in the pathogenesis of MH [24, 29]. We demonstrated that BA diameters were significantly larger in P_MH_ than in P_control_1_ and P_control_2_, which suggests that BA hypertrophy plays a key role in MH in pwCF. Interestingly, BA diameters were significantly smaller in P_control_1_, who suffered more severe morphological changes than P_MH_. Since severe morphological changes and only minor hypertrophy of BAs are present in patients without MH, severe morphological changes might not be an important risk factor for MH. On the contrary, the presence of less severe morphological changes and significantly larger BA in P_MH_ might indicate BA hypertrophy to be a more pivotal contributing factor to MH. These results are corroborated by the fact that female patients have significantly smaller BA diameters [24] and 58% of P_MH_ were females compared to only 35% of females in matched P_control_2_.

Routine chest CT in pwCF is carried out without contrast agents, thus rendering assessment and measurement of BA impossible, and BA diameters are not part of any scoring system for CF [19–21].

### Limitations

The retrospective observational character of the study is a clear limitation as it is highly prone to bias. We tried to minimize these effects by blinding readers to patient characteristics and by mixing the CT scans of P_MH_ and P_control_ for reading. Both readers were not involved in BAE and could therefore not identify images or patients. In addition, MH in pwCF is a rare complication and the number of patients enrolled in this study would be very difficult to obtain in a prospective study design. Another limitation is the difference in global Helbich score and in ppFEV_1_ between P_MH_ and P_control‑1_, with both parameters more severe in P_control_1_, indicating more severe disease. We aimed to minimize this effect by choosing a severity matched sub-control group P_control_2_ that was comparable in global Helbich score and ppFEV_1_. The results from this subgroup confirmed the findings. However, both control groups were slightly younger than the P_MH_ group possibly creating a bias in BA diameters as BA diameters increase with age [24]. Older patients with more advanced lung disease more commonly develop MH [3, 4]. Higher morphology scores would therefore have been expected in lobes with MH in the older P_MH_ when compared to the younger P_control_1_ and P_control_2_, which was not the case. On the other hand, the control groups comprised more male patients, with males having significantly larger BAs, which was not the case either [24].

Then, MH might have influenced the subscores “ground glass infiltrate” and “mucus plugging” as intraluminal blood might have been misinterpreted, but only seven of 23 CT scans were carried out on the day before or on the day of the MH itself and both subscores were within normal range. However, this uncertainty remains.

Another, albeit minor, limitation was the fact that due to the multicenter study design, CT protocols of P_MH_ differed according to the transferring hospital. Mostly, P_MH_ arrived with external medical imaging at our center. However, the diagnostic value of all CT scans met high-quality standards. Chest CT of control patients was carried out as CT angiography. We did not consider this difference to influence the assessment of structural changes of lung parenchyma enough to create significant bias.

## Practical conclusion

- The results of our study put into question the prevailing hypothesis of severe morphological changes in patients with cystic fibrosis (pwCF) preceding major hemoptysis (MH).
- The severity of morphological changes was not higher in patients with MH (P_MH_) nor in lobes with MH than in matched control patients without MH. However, lobes affected by MH had higher Helbich scores compared to lobes without MH, which is why chest computed tomography (CT) assessment on a lobar level might be a better tool when assessing for impending MH.
- Several subscores were significantly higher in control patients but no difference was found between lobes with MH and lobes without MH. However, the subscore “abscess/sacculation” might need special attention as it was higher in lobes with MH than in a severity-matched control group.
- Bronchial artery (BA) diameters were significantly higher in P_MH_ compared to the more severely affected control group and the severity-matched control group, indicating that BA plays a pivotal role in MH.
- The results of this study indicate that although morphological changes and MH both occur with older age and in the same lung region, they might not be linked but may be parallel processes due to chronic inflammation.
- Our data provide an argument that commonly used scores to evaluate morphological CT changes in pwCF are not able to assess MH, and other factors, e.g., hypertrophied BA, not represented and not measured in these scores, might be more suitable for assessing the risk for MH.
- Scoring would need to be adapted to a lobar level to provide important clinical information when estimating the risk for MH, as MH seems to be a rather localized process.
- Further prospective studies combining, e.g., lobar CT evaluation, the patient’s perception of frail lung regions, and the actual occurrence of MH might help to identify important risk factors for MH and possibly predict MH in pwCF.

## Data Availability

The data that support the findings of this study are available from the authors of this article but restrictions apply to the availability of these data, which were used under license for the current study, and so are not publicly available. Data are however available from the authors upon reasonable request and with permission of the different institutions where patients are affiliated. Contact corresponding author MD for data request.

## Abbreviations

BA: bronchial artery
BAE: bronchial artery embolization
CF: cystic fibrosis
CT: computed tomography
MH: massive hemoptysis
P_MH_: patients with massive hemoptysis
P_control‑1_: control group 1 patients without hemoptysis
P_control‑2_: severity-matched control group 2 patients without hemoptysis
pwCF: patients with cystic fibrosis
ssBACE: super-selective bronchial artery coil embolization

## Declarations

This multicenter study has been approved by the ethics committee of Aerztekammer Nordrhein according to § 15 registry number 2016/181 and by the ethics committee of Medical School Hannover, registry number 10245_BO_K_2022 who also waived the need for informed consent from all patients. All methods were carried out in accordance with relevant guidelines and regulations.

The supplement containing this article is not sponsored by industry.
